# Effects of intraperitoneal immunoglobulin therapy on behavior and cognitive functions in CLP-induced sepsis model in rats

**DOI:** 10.1186/cc11996

**Published:** 2013-03-19

**Authors:** F Esen, E Senturk, P Ergin Ozcan, G Orhun, S Gümrü, M Kaya, N Orhan, N Arican, F Aricioglu

**Affiliations:** 1 University of Istanbul, Turkey; 2 Marmara University Faculty of Pharmacy, Istanbul, Turkey

## Introduction

The present study investigated the effects of a single dose of intraperitoneal (i.p.) IgG and IgGAM administration on various behavioral alterations in a cecal ligation perforation (CLP)-induced sepsis model in rats.

## Methods

Female Wistar albino rats (200 to 250 g) were divided into five groups (*n *= 8): a naive Control group, a Sham operated group receiving conventional antibiotic treatment, a CLP group receiving CLP procedure and conventional antibiotic treatment, and IgG and IgGAM groups which were also applied 1 g/kg, i.p. IgG and IGAM therapy 5 minutes after the CLP procedure. Ten, 30 and 60 days after the surgery, animals underwent three behavioral tasks: an open field test to evaluate the locomotor activity, an elevated plus maze test to measure the level of anxiety, and a forced swim test to assess the possible depressive state. The results acquired from these tests were used to estimate the effect of immunoglobulin therapy on behavioral changes in CLP-induced sepsis in rats.

## Results

In the open field test, the CLP group showed a significant decrease in total squares passed on days 10 and 30. Similarly, total numbers of rearing and grooming were dramatically decreased in the CLP group in comparison with control and sham groups (*P <*0.005). In the elevated plus maze test, the number of entries to open arms decreased in the CLP group. In the forced swim test, there was a tendency for increase in immobility time in the CLP group, although the data were statistically insignificant. All of these values which were indicating the importance of behavioral alterations were improved on day 60. Immunoglobulin therapy prevented the occurrence of these behavioral changes. Especially, animals in the IgGAM group conserved the values quite near to those of the control group in measured parameters.

## Conclusion

Sepsis, even though it has been treated with conventional antibiotics, caused a negative effect on behavioral parameters. In this study, IgG and IgGAM treated animals in the presence of CLP did not show these behavioral changes. Therefore our results suggest that a single dose of i.p. IgG and IgGAM treatment, which was applied immediately after the sepsis procedure, prevents behavioral defects observed following sepsis.

